# A Software Engineering Framework for Reusable Design of Personalized Serious Games for Health: Development Study

**DOI:** 10.2196/40054

**Published:** 2023-03-06

**Authors:** Stéphanie Carlier, Vince Naessens, Femke De Backere, Filip De Turck

**Affiliations:** 1 Internet Technology and Data Science Lab Faculty of Engineering and Architecture Ghent University Ghent Belgium; 2 Interuniversity Microelectronics Centre Ghent Belgium

**Keywords:** serious game, health care, personalization, domain knowledge, framework, eHealth, cocreation

## Abstract

**Background:**

The use of serious games in health care is on the rise, as these games motivate treatment adherence, reduce treatment costs, and educate patients and families. However, current serious games fail to offer personalized interventions, ignoring the need to abandon the *one-size-fits-all* approach. Moreover, these games, with a primary objective other than pure entertainment, are costly and complex to develop and require the constant involvement of a multidisciplinary team. No standardized approach exists on how serious games can be personalized, as existing literature focuses on specific use cases and scenarios. The serious game development domain fails to consider any transfer of domain knowledge, which means this labor-intensive process must be repeated for each serious game.

**Objective:**

We proposed a software engineering framework that aims to streamline the multidisciplinary design process of personalized serious games in health care and facilitates the reuse of domain knowledge and personalization algorithms. By focusing on the transfer of knowledge to new serious games by reusing components and personalization algorithms, the comparison and evaluation of different personalization strategies can be simplified and expedited. In doing so, the first steps are taken in advancing the state of the art of knowledge regarding personalized serious games in health care.

**Methods:**

The proposed framework aimed to answer 3 questions that need to be asked when designing personalized serious games: Why is the game personalized? What parameters can be used for personalization? and How is the personalization achieved? The 3 involved stakeholders, namely, the domain expert, the (game) developer, and the software engineer, were each assigned a question and then assigned responsibilities regarding the design of the personalized serious game. The (game) developer was responsible for all the game-related components; the domain expert was in charge of the modeling of the domain knowledge using simple or complex concepts (eg, ontologies); and the software engineer managed the personalization algorithms or models integrated into the system. The framework acted as an intermediate step between game conceptualization and implementation; it was illustrated by developing and evaluating a proof of concept.

**Results:**

The proof of concept, a serious game for shoulder rehabilitation, was evaluated using simulations of heart rate and game scores to assess how personalization was achieved and whether the framework responded as expected. The simulations indicated the value of both real-time and offline personalization. The proof of concept illustrated how the interaction between different components worked and how the framework was used to simplify the design process.

**Conclusions:**

The proposed framework for personalized serious games in health care identifies the responsibilities of the involved stakeholders in the design process, using 3 key questions for personalization. The framework focuses on the transferability of knowledge and reusability of personalization algorithms to simplify the design process of personalized serious games.

## Introduction

### Background

Serious games have a primary objective other than pure entertainment as they seek to educate or train, among others [1,2]. In many domains of health care, serious games have shown positive effects and are increasingly used to motivate treatment adherence, reduce treatment costs, and educate patients and their families on a specific pathology [3-7]. For example, serious games are used for the treatment of mental health disorders, such as anxiety disorders or depression [5,8-10], physical [11-14] or cognitive rehabilitation [15-17], or the education of health professionals and patients [18-22]. Multiple reviews exist on this emerging field that state that serious games can be an effective tool for health, but the research remains in its infancy, limited by design and evaluation challenges [5,10,20,21,23-25].

One of these challenges is the long-term and continuous support of the targeted users. The user’s abilities will not only evolve but also based on the user’s current context, a different configuration or approach might be called for. Many researchers have indicated the need for a personalized approach in serious games, abandoning the *one-size-fits-all* approach [26-31]. Serious games often fail to sustain long-term retention and treatment adherence if gamification mechanisms are not adaptive and cannot dynamically reengage the user [32,33]. Users lose motivation and games become predictable. Although the primary objective of a serious game is not entertainment, it remains crucial that the game is entertaining to retain user engagement [34]. To create long-term engagement in serious games, a balance between challenge and skill, leading to a state of flow, must be achieved [35].

Games tailored to the user generally result in better performance outcomes for the user, making personalization a key aspect of a successful serious game [36-39]. Different terms, such as adaptability, adaptivity, personalization, contextualization, and customization, are used in the literature to indicate the tailoring of serious games to users. Sajjadi et al [26] introduced the overarching term *individualization* when no distinction between these concepts is necessary. Adapting a serious game to the needs of a user can be done at design time, before starting the game (ie, static personalization), or while playing the game (ie, dynamic personalization).

*Adaptability* is defined by multiple researchers as the possibility to change an environment based on the user’s changing needs, whereas *adaptivity* is the dynamic or automatic adjustment of game elements to the individual’s actions or performance [26,29]. *Personalization* is characterized by the, often automatic, adaptation of the game based on the profile or context information of a specific individual user, such as heart rate or age [26]. *Customization* can be seen as changing the system based on the needs of a user group or an individual user, manually or automatically, and is often related to changes in appearance and content [26,40]. Streicher and Smeddinck [29] considered personalization as a specific form of customization, while they saw adaptability and adaptivity as a means to achieve personalization or customization. These concepts refer to the tailoring of the game at run time (ie, before or during gameplay), which differs from the player-centered design in which decisions are made during the design of the game based on the needs of a specific target group [41].

Not only do people learn in different ways and paces and perceive the difficulty level of the game differently, but the game itself is also experienced differently by different people, and not all game elements will work for everyone. In addition, the specific skills of the user might vary and develop over the course of playing the game [26,35,42,43]. The game should therefore be able to respond by adapting to the user, that is, ensuring a state of flow [4,35].

**Figure 1:**
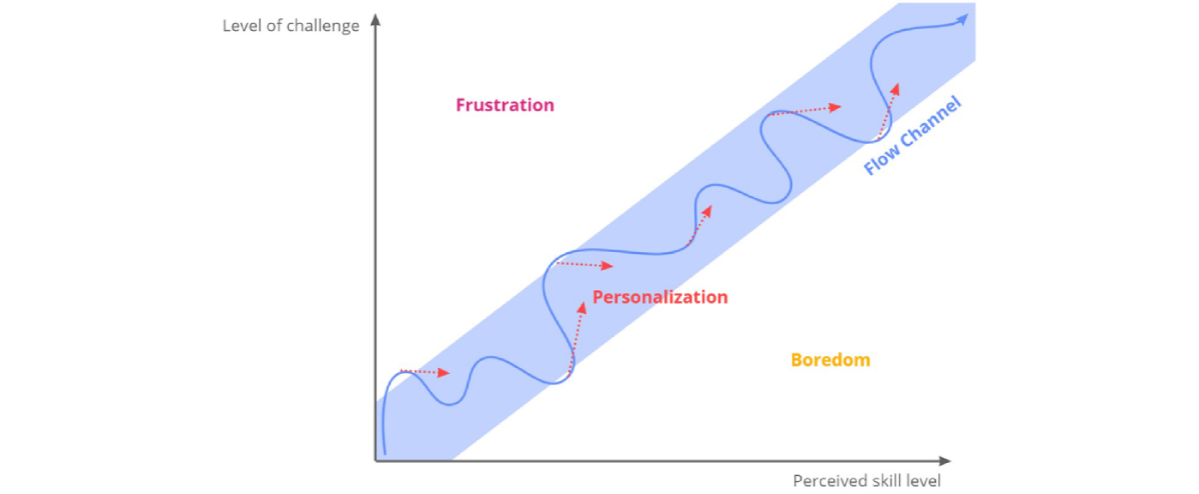
The Flow Model states that to enter the flow channel, that is, a state of total immersion and maximized focus and performance, the goal and related challenge should match the skill level of the user [46].

Flow theory models the relationship between the level of challenge in the game and the skill level of the user [44,45]. According to the Flow Model, the user is in a state of flow (ie, total immersion with maximized focus and performance) when the game has a clear goal and the user receives direct feedback on their performance related to this goal [45]. More importantly, to enter this state of flow, or the flow channel, the goal and related challenge level of the game should match the skill level of the user [4,29]. Frustration occurs when the game is too difficult for the perceived skill level and boredom sets in if the user is not challenged sufficiently by the game. Therefore, a serious game should maintain a balance between these parameters, even as the skill level of the users increases throughout the course of the game, to ensure that a state of flow is achieved, as shown in Figure 1. Because people differ and learn at different rates, the serious game should be able to detect and respond to the changing context and skill level of the user [4].

Another disadvantage of current gamified health applications is that the level of customization is often lacking, resulting in gamification that does not take health purposes, changes, or target groups into account [32]. Entertainment games already include different preferences among users by identifying player types using models such as the Bartle Model [46]. However, these models cannot be generalized to serious games because they are too limited. More context or player aspects need to be considered for the personalization of serious games, such as anxiety, stress, learning style, engagement, performance, skill level, and so on [26]. In contrast to the audience of entertainment games, the target audience of serious games is larger, including nongamer types.

Serious games and gamification, with a user-centered, adaptive, and personalized approach, show promise in increasing treatment adherence and boosting engagement with interventions [3]. Creating a personalized serious game from scratch for each type of user is a costly and challenging operation for developers and domain experts [17,29]. To create a serious game, expert knowledge of relevant domains is necessary. Often, therapists and domain experts are continuously involved in this design process in various ways [28,47]. Some examples are as follows: the involvement of a team of therapists in the design process of a serious game for anxiety reduction in children with autism spectrum disorder [8]; conducting in-depth interviews with occupational therapists for a serious game for cognitive impairment [15]; and validating the content of an informational serious game on COVID-19 by consulting a specialized team of physicians, professors, and medical students [48].

Different methods exist to include personalization in serious games. Some approaches focus on classifying the user according to a certain player type [41,49,50], whereas others focus on changing game aspects [33,35,51]. Rule-based adaptation defines rules that, when satisfied, lead to predefined actions that determine the further course of the game. However, this can lead to a less effective game because adaptation options are limited by the predefined rules [4]. Plan-based adaptation can be considered as a collection of state machines. Each plan is a state machine of which each state is an executable action in the game. If a condition is met, the active states can be selected to determine the course of the game. This adaptation method can, for example, be used in games containing several storylines, unlocking different aspects of the story depending on the user’s current physiological state (eg, relaxed vs stressed states) [4]. Model-based adaptation allows the creation of models for various game elements. These models can dynamically change based on the changing user information. This method allows for more complex analysis and adaptation techniques such as the use of artificial intelligence to predict the progression of the user in the future [4,52]. Studies exist on how gamification and serious games can be personalized and which factors influence these decisions [53]. However, it remains unclear how this information can be integrated into the design and implementation of a fully personalized and adaptive serious game.

Research exists on frameworks for the design of serious games; however, these are often designed for specific health care domains, such as physical [13,54-56] or cognitive rehabilitation [15,57,58], or focus on specific technologies, such as emotion recognition and gamification patterns [59,60]; only a few focus on the development of serious games in general [28,61]. Nevertheless, these frameworks often remain vague on how personalization can be achieved, use one specific model for personalization, or do not target personalized serious games [13,60,61]. Moreover, only one study reported the need for reusable serious games components and proposed a serious game framework for reusable intelligent software components (eg, emotion recognition and learning algorithms) [62]. However, this study is limited to the implementation of software components and does not state how domain knowledge and intelligent personalization algorithms can be integrated into the multidisciplinary design process of serious games.

Owing to the lack of standardized frameworks for the design of a (personalized) serious game, the challenging nature of the design process, and the focus on using case-specific guidelines, the evaluation of serious games is often limited and varies widely as different approaches are taken. Research should aim to evaluate the effectiveness of serious games more rapidly and in a controlled setting, focusing on comparing approaches using the same environment [10,17].

### Objective

Overall, when creating a personalized serious game, 3 questions need to be asked:

- Why is the game personalized?
- What parameters can be used for personalization?
- How is the personalization achieved?

In this study, we aimed to answer these questions by proposing a software engineering framework that streamlines the design process of personalized serious games and facilitates the reuse of domain knowledge and personalization algorithms to reduce development costs.

**Figure 2:**
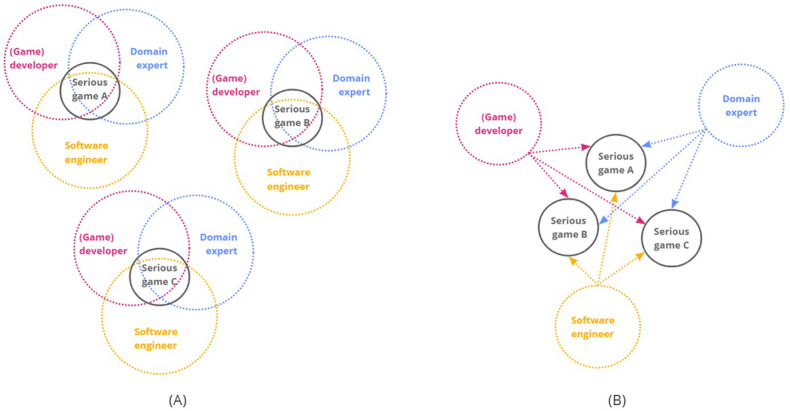
(A) A conceptual schematic visualization of the currently complex and redundant dependencies between stakeholders during the development of multiple personalized serious games. (B) Schematic visualization of decoupling these dependencies by implementing reusable components that can be used for multiple serious games, thereby removing the tedious and repetitive effort of the stakeholders.

Currently, the development of personalized serious games is a costly and complex process that requires the continuous involvement of different stakeholders, such as (game) developers, domain experts, and software engineers. Furthermore, this process must be completely repeated for each serious game and is often use-case specific. This means that each stakeholder should share their expertise throughout the design process of each new serious game, irrespective of the subject of the game, as their knowledge is never formalized or transferred to new serious games. This results in a complex process with many dependencies and redundancies between different stakeholders and serious games, which can be visualized as shown in Figure 2A. This approach complicates the evaluation of serious games and personalization strategies. With that many different parameters, such as design approach, target audience, or personalization algorithms, the comparison and evaluation of different personalization strategies are limited. Moreover, very little is known about how personalization strategies can be applied to serious games, as research is focused on the development of serious games for specific use cases, without attention to reusability.

This study aims to reduce these disadvantages by proposing a framework to simplify this process. The framework streamlines the development process of personalized serious games by considering the value of cocreation with different stakeholders, as shown in Figure 2B. However, by focusing on the reusability and formalization and transferability of expert knowledge, the dependencies between the stakeholders can be decoupled, thereby reducing the development cost of a personalized serious game.

The following paragraphs are structured as follows. The *Methods* section describes the *Use and Design of the Software* engineering framework, followed by the *Generic Framework,* which discusses the software engineering framework and the responsibilities of the involved stakeholders. To assess the proposed framework, a simple proof of concept was implemented, that is, an existing game was first transformed into a serious game, which is explained in the *Methods* section in *A Serious Game for Shoulder* *Rehabilitation.* Next, the possibilities of the framework were illustrated by transforming this framework into a personalized serious game in the section *Proof of Concept: an Adaptive and Personalized Serious* *Game,* followed by a discussion of the evaluation of the resulting serious game in the *Results* section. Finally, the conclusions of this study are discussed in the *Discussion* section.

## Methods

### Use and Design of the Software Engineering Framework

The proposed framework provides a software engineering perspective on how the design process of serious games can be made more efficient by focusing on personalization and reusability. It can be used to transform existing (serious) games into personalized serious games as well as when designing new personalized serious games. As the proposed framework is a software engineering framework, it acts as an intermediate step between conceptualization and implementation, thereby aiming to close the gap between serious game design and implementation.

The Mechanics, Dynamics, and Aesthetics framework is widely accepted in game design as a formal approach for conceptualizing the dynamic behavior of game systems [63]. It approaches a game from the perspective of the player and discerns mechanics (ie, the actions, goals, and rules of the game), dynamics (ie, the behavior followed by the player’s interaction with the mechanics), and the aesthetics (ie, the desired emotional responses of the player when playing the game). The Mechanics, Dynamics, Aesthetics, and Outcomes (MDAO) framework is an extension of the Mechanics, Dynamics, and Aesthetics framework for the conceptualization of serious games [64], and it introduces the concept of outcomes, that is, the behavioral or intellectual responses of the player after playing the game. The MDAO framework approaches a game from the player’s perspective by first defining the outcomes and aesthetics, followed by dynamics and mechanics. To define the necessary concepts, a domain expert (outcomes and aesthetics) and a game developer (dynamics and mechanics) are needed. The results of the MDAO framework can then be used in the process of answering the 3 questions the framework poses: Why is the game personalized? What parameters are used for personalization? and How is personalization achieved?

Furthermore, the proposed framework is an extended version of the adaptive experience engine proposed by Bellotti et al [65]. They proposed an architecture that decouples the content of educational tasks from the game aspects in sandbox serious games, that is, games that encourage free play, to standardize the development of educational serious games and increase the efficiency by focusing on the reusability of educational tasks. The adaptive experience engine of Bellotti et al [65] uses educational content or tasks, defined by pedagogical experts, and stores these tasks in a common repository for reuse in different games. A game author is then responsible for specifying the requirements of the delivery of such a task at run time, that is, determining the type of task and time it is relevant during gameplay.

We proposed an extension of this framework for the personalization of serious games for health (SGH). We defined the necessary building blocks to increase the reusability of the different components and assign stakeholder responsibilities to these components. Instead of a repository of educational tasks, a knowledge base, which was designed according to the requirements of the domain experts, was included to model the necessary domain knowledge of a serious game. Furthermore, a personalization engine allows for the inclusion and evaluation of multiple personalization algorithms. The proposed framework was evaluated by implementing a proof-of-concept serious game to validate that the framework meets the expectations of the authors.

### Generic Framework

#### Overview

**Figure 3:**
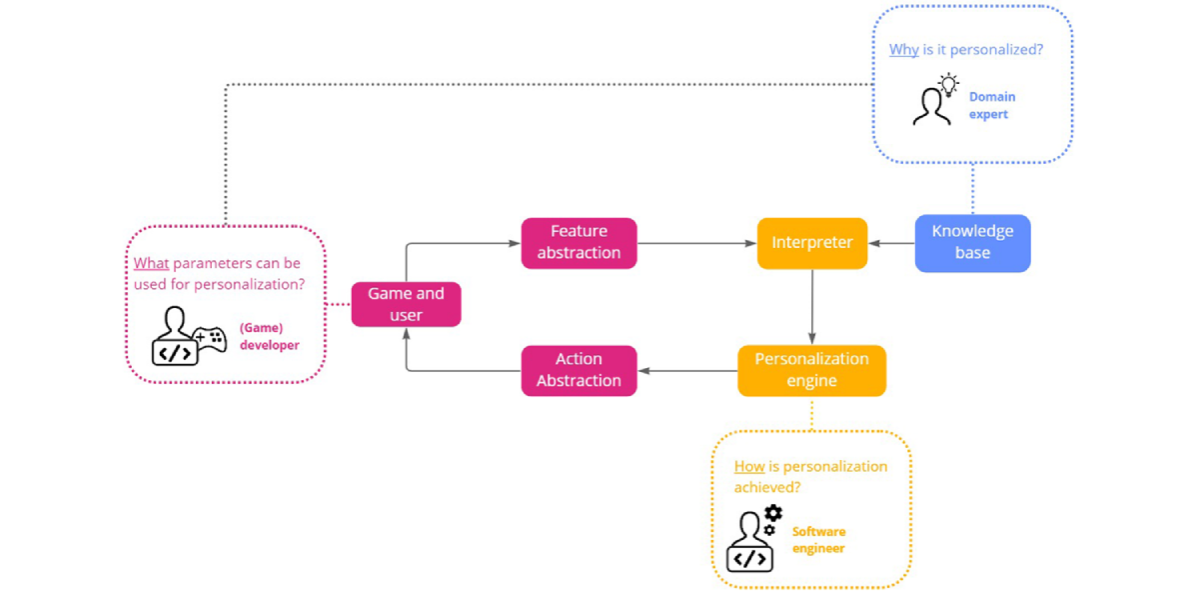
The generic framework consists of 3 types of modules. The Knowledge Base module formalizes the knowledge of the domain expert. The game-specific modules are the responsibility of the (game) developer and contain the personalized parameters. The final module, the Independent Personalizer, is the responsibility of the software engineer, who implements the algorithms for personalization.

The framework decoupled the domain knowledge from the personalizable variables in the serious game and personalization algorithms. This allowed the modification or addition of expert knowledge and game concepts independently of each other and the exploration of different personalization strategies without altering the structure of the game itself. The generic architecture discerned 6 modules through which a personalization loop flowed, moving through the different modules to translate user and game data into a specific knowledgeable game task that resulted in the adaptation of the game, as shown in Figure 3.

#### Domain Knowledge

The *Knowledge Base* module is at the heart of the framework and aims to answer the question, *Why is there a need for personalization?* More specifically, this module is responsible for modeling the knowledge of the domain experts that gives insight into the serious aspect of the game. Serious games are defined as games that have objectives other than pure entertainment. For example, in the case of a rehabilitation scenario, this component will contain certain information regarding rehabilitation exercises and the conditions under which they can be executed, for example, injury type, skill level, and rehabilitation progress. For this example, the expert knowledge would indicate that personalized support during rehabilitation is necessary to accommodate different injury types, different phases of the rehabilitation process, skill levels, or more, thereby answering why there is a need for personalization. Such expert knowledge can be modeled or computerized using different approaches, for example, simple approaches such as databases or functions or more complex constructs such as ontologies. The goal of the *Knowledge Base* module is to allow the transferability of knowledge, and the module can thus be reused or replaced based on the domain in which the serious game is situated.

#### Game-Specific Modules

The 3 modules are game specific, namely, the *Game and User* module, which contains the game itself, and the *Feature Abstraction* and *Action Abstraction* modules, which function as a layer of abstraction between the game and the rest of the system. In consultation with the domain expert, the (game) developer can identify the necessary parameters for personalization, answering the question, *What needs to be personalized?* Two sets of parameters, namely, features and actions, are discerned at design time. First, the information that triggers personalization is defined as a feature. These features can be game information, such as scores, or physiological information collected using wearables (eg, heart rate). These features, which trigger the personalization, introduce a feedback loop in the system to ensure that it is adapted according to the needs of the user. For example, when a score or heart rate is too high, the game might be too exhausting, which should result in the adaptation of the system. In addition, when the user continues to score badly, the game might be too difficult, which in turn will result in the adaptation of the difficulty level. Second, the actions are a set of game parameters that are personalizable based on the context information, such as speed and difficulty level, or more complex constructs, for example, to personalize a storyline. At run time, the features are then periodically sent to the *Feature Abstraction* module, where they are abstracted to a generic format that can be interpreted by the game-independent modules. Existing player-type frameworks, such as the Hexad Framework, can be used to identify the parameters for personalization [64].

#### Independent Personalizer

Two modules, namely, the *Interpreter* module and the *Personalization Engine*, are game and domain independent and can thus be replaced or reused for the personalization of new or existing games. The software engineer aims to answer the question, *How is personalization achieved?* in these modules without the need to interact with the game itself. The *Interpreter* module interacts with the *Knowledge Base* and the game-specific modules to fetch the necessary information to understand the data it has just received. The module then interprets and translates the data into a format that can be understood using the *Personalization Engine*. This value contains the necessary information for the *Personalization Engine* to determine whether personalization is necessary and the degree of personalization without the need for context information. For example, if the user has an extremely high heart rate in a relaxation game, the *Interpreter* will understand, based on the context and domain knowledge that it receives, that the user’s heart rate needs to be lowered and personalization has to be applied accordingly. Next, the *Personalization Engine*, containing one or more models for personalization, applies the action and sends it to the *Action Abstraction* module, a game-specific module that knows the game task that this action maps (eg, changing the speed of the game). In turn, the *Action Abstraction* module sends a game-specific task order to the game, which can then adapt accordingly and complete the personalization loop.

Different approaches for personalization exist, for example, intelligent algorithms such as reinforcement learning or recommender systems. By decoupling the personalization task from the game and domain knowledge, opportunities to explore the utility of different personalization models arise. Within one game, different approaches can be used to process or compare triggers for personalization. Furthermore, existing models can be reused for the development of new serious games, which simplifies and reduces the cost of creating a serious game.

### A Serious Game for Shoulder Rehabilitation

In physical rehabilitation, where patients need to repeat exercises regularly to train their mobility or balance, serious games can provide a welcome distraction from the repetitiveness of the treatment. Patients often lack the incentive to complete the time-consuming exercises at home or start rushing through, resulting in a decline in progress and exercise completion and once again demotivating the patient to correctly adhere to the treatment. Serious games for physical rehabilitation aim to motivate patients to increase their treatment adherence and effectiveness.

As a proof of concept, an existing game was transformed into a serious game and then used to illustrate how the proposed framework can be used to personalize existing serious games as well as when designing new serious games. The game was simple and contained a character that could be controlled by pressing a single button to make the character move upward and avoid upcoming obstacles, similar to the well-known game Flappy Bird (Gears). For this research, the game was transformed into a shoulder rehabilitation exercise that was suitable for physical rehabilitation after injury. As mentioned previously, in physical rehabilitation, it is important for patients to perform their exercises regularly and correctly.

**Figure 4:**
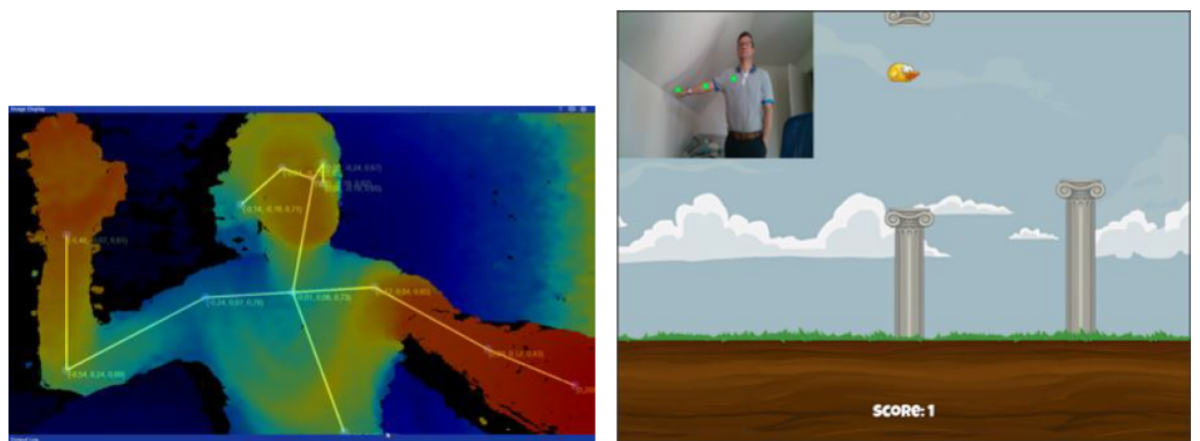
Using the Intel RealSense Camera and the Cubemos skeleton tracking (left), the arm movements of the user are tracked to control the game character (right).

The mechanics of the chosen game set the users up for failure, as they must continue controlling the character until it hit an obstacle and the game was over. This seems contradictory, as rehabilitation patients should not be rushed when performing their exercises, and this thus indicates the need for personalization by adapting the difficulty of the game to the capabilities of the user. Instead of controlling the character with a button, the user could control it by lifting their outstretched arm to the shoulder level and moving the character upward. The Intel RealSense Camera (Intel) [66] and the Cubemos skeleton (Intel) tracking SDK [67] were used to track the movement of the arm of the user and control the upward movement of the bird, as shown in Figure 4. The user earned a score based on how long they manage to keep the bird in the air without hitting any obstacles; otherwise, the game ended.

### Proof of Concept: an Adaptive and Personalized Serious Game

**Figure 5:**
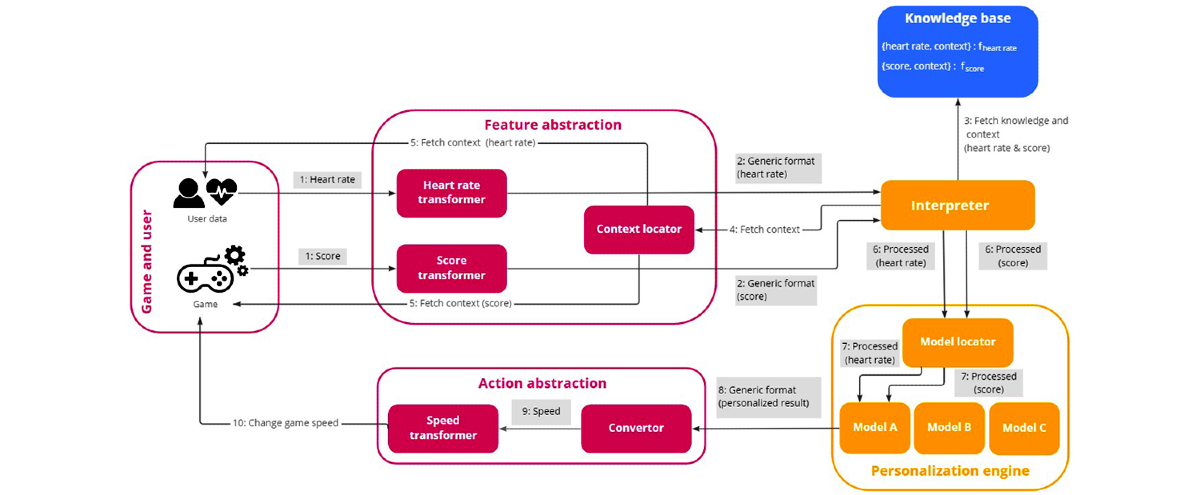
The implementation of the proof of concept using the proposed framework indicates that 2 features have been identified, namely, heart rate and game score. The Knowledge Base contains the necessary expert knowledge and respective context parameters that are necessary to interpret these features. After the Interpreter has interpreted this information, using the Context Locator to fetch the context values, the Personalization Engine is responsible for the adaptation, using the implemented models. Finally, this is again translated to an action of the game, namely, speed.

The following paragraphs discuss the different components of the generic framework in detail, using the implementation of the serious game for shoulder rehabilitation as an illustration of how the framework works. Figure 5 provides an overview of the implemented proof of concept using the proposed framework.

#### Domain Knowledge

As mentioned in the section *Generic Framework*, the *Knowledge Base* models the knowledge that is relevant for the specific serious game. This can be achieved using a simple database, storing values, or more complex constructs such as ontologies that are capable of modeling complex relationships between concepts in a computer-readable format. The responsibilities of the domain expert consist of (1) defining or reusing the necessary domain knowledge and, together with the (game) developer, (2) identifying the personalizable parameters of the serious game, that is, features and actions.

For this proof of concept, the *Knowledge Base* was kept simple. The *Knowledge Base* stored 2 functions, one for each identified feature, namely, f_heart_rate_ and f_score_. The function f_heart_rate_ calculated the highest accepted heart rate for the given context information, whereas the function f_score_ looked at previous N scores, given the necessary context to evaluate the performance of the user. This means that, based on the game and its objective, 2 features were identified, namely, heart rate and game score.

#### Game-Specific Modules

As previously mentioned, for the game, 2 features were identified, namely, heart rate and game score. These data were collected by the *Game and User* component and were used to trigger the start of the personalization loop as they were periodically sent to the *Feature Abstraction* module, which sent them to the independent personalization modules for evaluation. As patients cannot rush their rehabilitation exercises but should perform them correctly to increase their mobility, the speed of the game was identified as a personalizable action. If the heart rate of the user increased or their game score decreased, the speed of the game should be lowered to continue to ensure good shoulder exercise performance.

Personalization of the game can occur both online (ie, in real time) and offline. For offline personalization, the feature data were only sent after a gaming session, adapting the game for the next session, whereas for real-time personalization, the game was personalized during the gaming session, based on the data received up to that point. One game session was considered to be a level of the game. Both types of personalization have their benefits: real-time personalization allows the game to quickly respond to the user’s currently changed context, whereas offline personalization facilitates a more complex analysis that considers the overall performance and progress of the user, instead of just a moment in time.

For each feature, the *Feature Abstraction* module contains its respective *Transformer* module, responsible for translating the data to a generic format, which can be interpreted by the independent personalization modules. Using this generic format, the features can be interpreted by the rest of the system without the need for game-specific information. Nonetheless, these features had very little meaning without the necessary context information. For example, it was difficult to interpret a heart rate of 100 without any additional information. However, given the knowledge that this was the resting heart rate of a 25-year-old male, it was possible to interpret the importance if this heart rate. Therefore, the *Context Locator* module was responsible for providing the relevant context information to the personalization modules when needed. The *Context Locator* knew which context mapped to what features and fetched this information from the *Game and User* module.

This decoupling reduced the integration of a new serious game for personalization to the following steps: (1) identifying the features, (2) identifying the actions, (3) implementing or reusing the respective *Transformer* modules, and (4) creating a *Context Locator* that fetched the relevant context information for the correct feature.

#### Independent Personalizer

After the *Interpreter* receives the feature data in a generic format, it contacts the *Knowledge Base* to fetch the information to interpret the received value. This information includes the expert knowledge and context parameters that are linked to a specific feature. For the game, the knowledge linked to the heart rate feature was f_heart_rate_ and the relevant context parameters were age, sex, and game intensity. For the score feature, the linked knowledge was f_score_ and the relevant context parameters were the previous N scores.

Next, the *Interpreter* module contacts the *Context Locator* to fetch the values of these context parameters for each feature. After receiving all the required information, the *Interpreter* can give meaning to the feature data and transform it into a processed value that contains the necessary information for the *Personalization Engine*, that is, the degree of the needed personalization, but is devoid of any game- or domain-specific knowledge. Continuing the example, the feature data, a heart rate of 100, and the associated context, the resting heart rate of a 25-year-old male, can be interpreted as an unusually high heart rate that should be reduced.

The *Personalization Engine* receives this processed value, and its *Model Locator* sends it to the correct model. As mentioned in the previous section, multiple types of models exist; however, for the proof of concept, only simple rule-based models were implemented to illustrate the framework. The first model receives the processed score as input and is used as an offline adaptation model, that is, the score after each gaming session is processed and used for the adaptation of the speed for the next gaming session. The second model takes the processed score and heart rate as input and is used for offline adaptation, whereas the third model receives the same input but is used for real-time adaptation.

The output of these models is a personalized value that is sent to the *Action Abstraction* module. This module, more specifically, the *Speed Transformer*, performs the reversed action of the *Feature Abstraction* module, as it transforms this generic format into a game-specific format, namely, to an action of the game, that is, speed.

**Figure 6:**
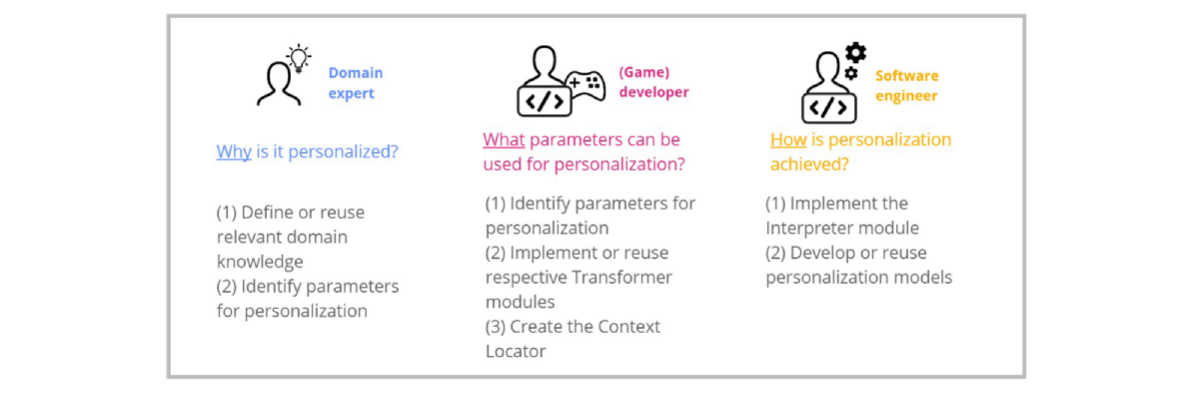
An overview of the responsibilities of the involved stakeholders.

As the *Interpreter* processes these input values, and the *Action Abstraction* module processes the output values, the software engineer does not have to have any domain- or game-specific knowledge to implement personalization algorithms. This reduces the responsibilities of the software engineer in (1) implementing the *Interpreter* module and (2) developing or reusing personalization models. Figure 6 presents an overview of the responsibilities of each of the involved stakeholder.

## Results

### Simulation of Game Scores

To evaluate the proposed framework, the implementation of a personalized game is assessed using several simulations. First, the response of the system was evaluated when only game scores were used as features. Each score represents the score obtained after a single game. The difficulty level at the start of a gaming session was always 0.8. Adaptation of the speed would only occur after a completed game, that is, offline personalization.

**Figure 7:**
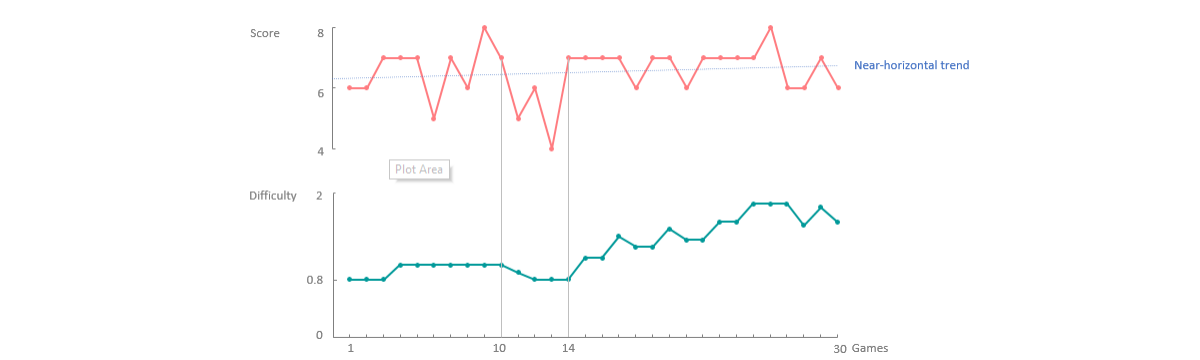
The first 10 games show a diverging score (top), which responds to a near-constant difficulty (bottom). The score of the user drops as of game 10, to which the system responds with a drop in difficulty. After game 14, the user achieves, on average, a constant score, which is, as expected, responded to by the system with a slow increase in difficulty.

The first series of scores was simulated to show a near-horizontal trend; that is, the user achieved, on average, a constant score for each game. The system used the previous scores of the user to decide whether the difficulty, that is, the speed of the bird, must be personalized. Thus, the difficulty was expected to be increased if this trend continued. Figure 7 shows the simulated scores that were fed to the system (top panel) and the response of the system regarding the adaptation of the difficulty (bottom panel). The gaming scores from the first 10 games showed a more diverging scoring pattern, indicating some drops in the score, and the system responds accordingly by lowering the difficulty. Game 14 showed a substantial increase in the gaming score, followed by a nearly constant score. As shown in the figure, the system reacted to this trend as expected by continuously increasing the difficulty of the game.

**Figure 8:**
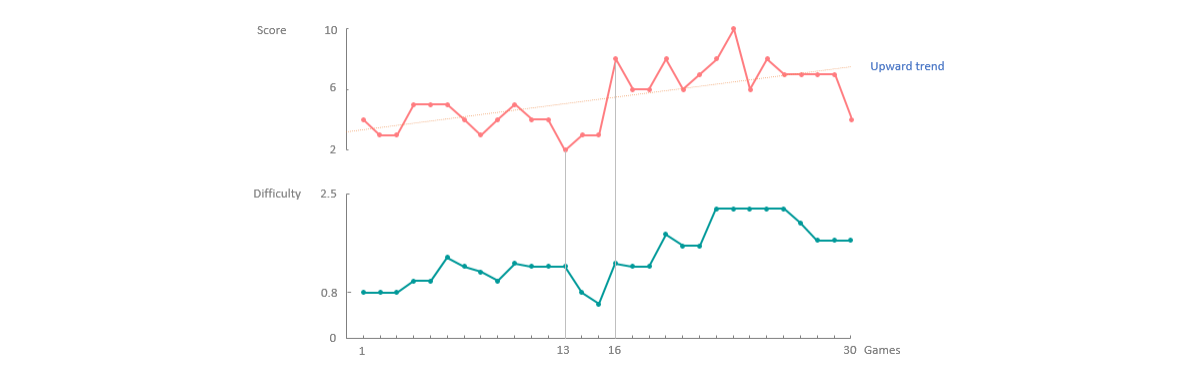
The score of the user is, on average, constant during the first 13 games (above), to which the system responds with a slight increase in difficulty (below). After a brief drop in scores, the user’s score indicates an upward trend as of game 16. The system responds by significantly increasing the difficulty of the game as long as this upward scoring trend continues.

A second simulation showed continuously improving game scores, that is, on average, upward trend, as illustrated in Figure 8. As the user continued to improve their previous score, the game was most likely to become too easy for the user. The expected response of the system was to increase the difficulty of the game more visibly during this upward trend than during a nearly constant trend. As of game 16, after a brief drop, the user significantly improved their game scores, upholding an upward trend. As expected, the system countered this by increasing the difficulty significantly faster than during the first 13 sessions, when the user achieved a constant score.

**Figure 9:**
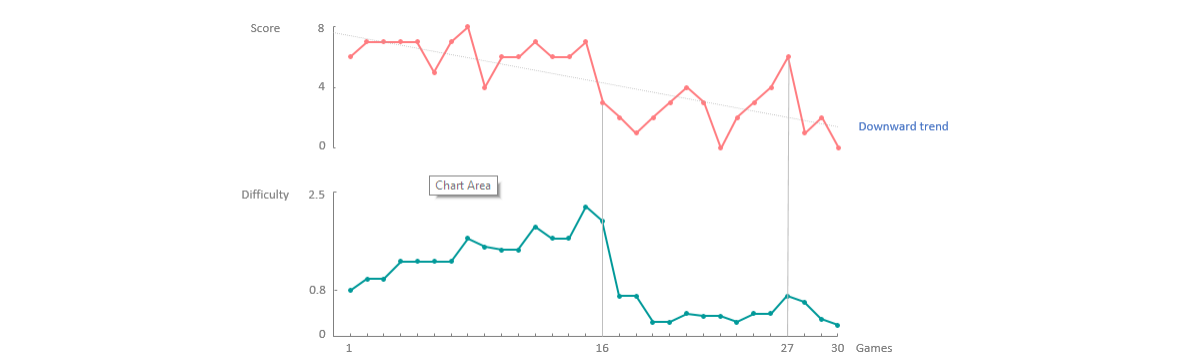
In the first 15 games, the user reaches, on average, a constant score (above), to which the system again responds by slowly increasing the difficulty (below). As of game 16, the score keeps dropping, showing a downward trend, to which the system responds by significantly lowering the difficulty.

The final simulated game series illustrated the situation in which the performance of the user continued to drop, that is, a downward trend in the game scores, as illustrated in Figure 9. Such behavior might indicate that the game is too difficult for the user and the game should respond by significantly decreasing the difficulty. As of the 16th game, the score of the user kept dropping, to which the system responded, as expected, by decreasing the speed of the game. By game 27, the user reached a new local maximum, which was answered by the system by again a slow increase in difficulty.

### Simulation of Game Scores and Heart Rate

For a second evaluation of the system, game scores and a rising heart rate, which at some point would exceed the set maximum heart rate of 180, were simulated. For this particular use case, where the user should perform a simple arm exercise, an elevated heart rate could indicate that the patient was distressed or experiencing pain, which would negatively affect the obtainable goal, that is, increasing the mobility of the arm. Therefore, the system should respond to this elevated heart rate by adapting to the game. The response of the system was compared for both offline, that is, after a gaming session, and real-time personalization, that is, during a gaming session.

For this simulation, we expected that a high heart rate would have a greater impact on the personalization of the game than the gaming score. If a rising heart rate was detected and the heart rate approached the maximum threshold heart rate of 180, the system should respond by immediately reducing the speed of the game. However, for offline personalization, the system was only able to respond if the average heart rate of the last completed game session exceeded 180. Therefore, the system could only reduce the speed of the game after a completed game session. For real-time personalization, the system was expected to respond much sooner and decrease the speed of the game from the moment the real-time heart rate exceeds the threshold value during the gaming session.

**Figure 10:**
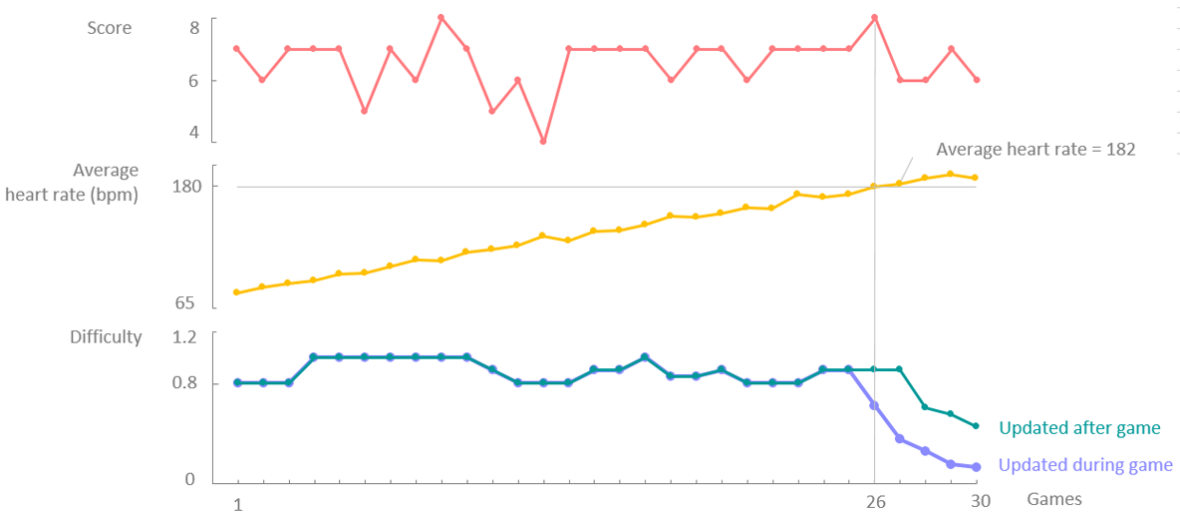
For an, on average, constant score (above) and a constantly increasing heart rate (middle), the system responds differently for real-time personalization compared with offline personalization (below). If the system is updated after the game (offline personalization), the maximum threshold heart rate of 180 bpm is exceeded in game 27, of which the average heart rate is 182 bpm. The system thus starts decreasing the difficulty as of game 28. When the system is updated during the game (real-time personalization), the system already decreases the difficulty as of game 26, therefore achieving a much lower speed much faster. bpm: beats per minute.

For this evaluation, the constant gaming score from the first simulation (Figure 7) was reused. Figure 10 illustrates the simulated gaming score (above), simulated average heart rate per gaming session (middle), and response of the system for both offline adaptation and real-time adaptation (below). For offline adaptation (green), the difficulty during a gaming session remained constant and was adapted only after the game has been completed. For real-time adaptation (purple), the difficulty could vary during a gaming session as real-time adaptations occurred. The difficulty values illustrated in Figure 10 correspond to the difficulty level at the end of each gaming session.

In the first simulation of a constant game score, the expected behavior was an increase in the difficulty. However, the system then also had to consider a rising heart rate, which resulted in more or less constant difficulty as long as the heart rate continued to rise. For offline adaptation, as soon as the average heart rate of a game session crossed the threshold value, which was the case for game 27, the system responded by decreasing the difficulty. Because offline adaptation introduced a certain delay, the game could only respond by game 28. The figure shows that this delay was not visible in the case of real-time adaptation, as the difficulty had already decreased for game 26.

**Figure 11:**
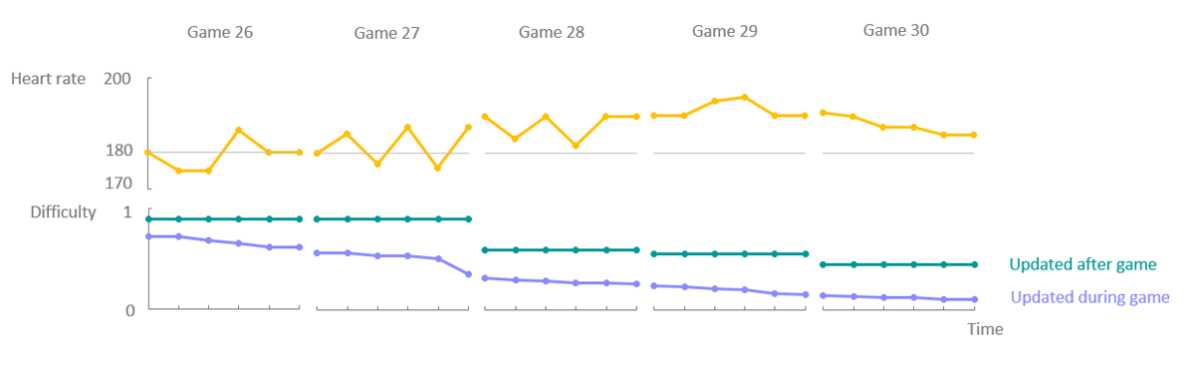
This detailed overview of the heart rate of the user starting from game 26 (top) indicates that the system can reduce the difficulty of the game (bottom) much faster in the case of real-time adaptation as the maximum threshold of 180 is already exceeded in game 26. Because the average heart rate of a gaming session only exceeds 180 in game 27, a delay is introduced in the version using offline adaptation. Real-time adaptation, therefore, allows the system to respond much faster to critical values than when offline personalization is used.

Figure 11 provides a detailed overview of how the system reacted (below) to the real-time heart rate (above) during games 26 to 30. Here, it is clear that the user’s heart rate already exceeded the threshold value during game 26, and thus the system could immediately reduce the difficulty of the game in the case of real-time adaptation. Because of the rigidity of offline adaptation, that is, one set speed for an entire game, the difficulty also decreased more slowly compared with real-time adaptation. The minimum difficulty for offline personalization of the entire exercise session was reached in game 30, whereas this minimum was already reached in game 27 using real-time personalization, reaching an even lower speed by the end of game 30.

## Discussion

### Principal Findings

This study proposes a framework to standardize and simplify the design of personalized SGH. The process of designing a serious game is challenging and involves multiple stakeholders. The proposed framework identifies the responsibilities of the involved stakeholders using 3 key questions. The framework focuses on the reusability, formalization, and transferability of the expertise of these stakeholders to simplify this process. As a proof of concept, a simple game was transformed into a serious game for shoulder rehabilitation. The game was implemented according to the proposed framework to illustrate how it can streamline the design and implementation of personalized serious games. Several simulations were conducted to evaluate the personalization mechanics of the resulting personalized serious game.

The integration of the framework introduces several advantages for the design of personalized serious games. First, by assigning responsibilities to the stakeholders involved, the cocreation of the serious game is still respected, but the complicated process is simplified. Each stakeholder knows what is expected of them and with whom they need to communicate to receive specific information. By introducing 3 types of modules, namely, domain knowledge, game-specific modules, and an *Independent Personalizer*, and defining the communication between these modules, the reusability of these modules becomes possible, introducing a sort of “plug-and-play” structure of the components. Domain knowledge can be formalized using complex structures, such as ontologies or a simple information database, and can be reused or extended to multiple serious games. Over time, researchers can gather an extensive knowledge base covering one or more domains, each time reducing the effort needed to integrate their knowledge into a new serious game. A similar approach can be adopted for the other modules.

Furthermore, this framework allows for the integration of multiple personalization algorithms or approaches. As discussed in the *Introduction* section, different methods exist for the personalization of serious games, but very little is known about their effectiveness. Using this framework, software engineers can focus on further developing and evaluating these personalization approaches without the need to repeat the entire process from scratch. Thus, more effort can be directed toward developing more complex and intelligent algorithms. However, the framework allows not only the reusability of algorithms but also multiple algorithms to be used simultaneously for one serious game, as the *Personalization Engine* can contain multiple models for the identified features. This can be interesting when both real-time and offline personalization needs to be considered. Serious games in the health care domain often deal with gathering time-sensitive information, collecting health sensor data, or monitoring the performance of the user, and wrong movements can have detrimental effects. The framework allows the integration of specialized personalization models that monitor these data and intervene instantaneously when needed without the need to interrupt the rest of the personalized game mechanics, as these models can exist independently of one another.

Finally, the literature has shown that the evaluation of the effectiveness of serious games is often inadequate because of sparse evaluation results, lack of standardized evaluation approaches, and high cost of designing serious games. This framework takes the first steps toward a more accessible and standardized evaluation procedure for serious games. The framework easily allows the control of different parameters by interchanging the components under evaluation, such as personalization algorithms, and reusing other components. Moreover, because of the reusability of components, different personalization strategies for serious games can be tested and evaluated at a much faster pace.

### Future Work

Although this study offers many contributions, it has some limitations that will be addressed in future work. First, the complexity of the proof of concept is limited to illustrating the functioning of the framework. Second, the resulting personalized serious game was evaluated using simulations. Therefore, future work will focus on implementing a more complex knowledge base and intelligent algorithms. Using this framework will facilitate the evaluation and comparison of different artificial intelligence algorithms, such as recommender systems or machine learning algorithms. The resulting serious game will be assessed using large-scale evaluations with end users to ensure that the personalization strategies used fit the needs of the target audience. To this end, questionnaires such as the System Usability Scale [68] and the Gameplay Scale [69] will be essential for thorough evaluation.

### Conclusions

We proposed a framework for the design of personalized SGH. The aim of this framework is to offer guidelines that streamline the complex cocreation process of serious games. Moreover, the implementation of this framework facilitates the transferability of domain knowledge and reusability of personalization algorithms, thereby taking the first steps to the state of the art concerning personalization in SGH. Complex models of specific domain knowledge can be reused and extended, while different personalization strategies can be easily compared and evaluated.

## Abbreviations

MDAO: Mechanics, Dynamics, Aesthetics, and Outcomes
SGH: serious games for health
